# Impulsivity in Binge Eating Disorder: Food Cues Elicit Increased Reward Responses and Disinhibition

**DOI:** 10.1371/journal.pone.0076542

**Published:** 2013-10-16

**Authors:** Kathrin Schag, Martin Teufel, Florian Junne, Hubert Preissl, Martin Hautzinger, Stephan Zipfel, Katrin Elisabeth Giel

**Affiliations:** 1 Department of Psychosomatic Medicine and Psychotherapy, University Hospital Tübingen, Eberhard Karls University, Tübingen, Germany; 2 fMEG-Center, University Hospital Tübingen, Eberhard Karls University, Tübingen, Germany; 3 Institute of Medical Psychology and Behavioral Neurobiology, Eberhard Karls University, Tübingen, Germany; 4 Department of Psychology, Clinical and Developmental Psychology, Eberhard Karls University, Tübingen, Germany; Paris Institute of Technology for Life, Food and Environmental Sciences, France

## Abstract

**Background:**

Binge eating disorder (BED) represents a distinct eating disorder diagnosis. Current approaches assume increased impulsivity to be one factor leading to binge eating and weight gain. We used eye tracking to investigate both components of impulsivity, namely reward sensitivity and rash-spontaneous behaviour towards food in BED for the first time.

**Methods:**

Overweight and obese people with BED (BED+; *n* = 25), without BED (BED−; *n* = 26) and healthy normal-weight controls (NWC; *n* = 25) performed a free exploration paradigm measuring reward sensitivity (experiment 1) and a modified antisaccade paradigm measuring disinhibited, rash-spontaneous behaviour (experiment 2) using food and nonfood stimuli. Additionally, trait impulsivity was assessed.

**Results:**

In experiment 1, all participants located their initial fixations more often on food stimuli and BED+ participants gazed longer on food stimuli in comparison with BED− and NWC participants. In experiment 2, BED+ participants had more difficulties inhibiting saccades towards food and nonfood stimuli compared with both other groups in first saccades, and especially towards food stimuli in second saccades and concerning sequences of first and second saccades. BED− participants did not differ significantly from NWC participants in both experiments. Additionally, eye tracking performance was associated with self-reported reward responsiveness and self-control.

**Conclusions:**

According to these results, food-related reward sensitivity and rash-spontaneous behaviour, as the two components of impulsivity, are increased in BED in comparison with weight-matched and normal-weight controls. This indicates that BED represents a neurobehavioural phenotype of obesity that is characterised by increased impulsivity. Interventions for BED should target these special needs of affected patients.

## Introduction

Binge Eating Disorder (BED) represents an empirically validated eating disorder [1]–[3] that is now a distinct eating disorder in the new release of the diagnostic and statistical manual of mental disorders (DSM-5) [4], [5]. The core psychopathology of BED according to DSM-IV and DSM-5 are binge eating episodes [5], [6]. These are characterised by an unusually large food intake within a discrete period of time while experiencing loss of control. With a lifetime prevalence of 1–3%, BED is the most common eating disorder in the general population and is often associated with overweight and obesity [7], [8].

Nevertheless, research relating to underlying mechanisms of BED is sparse. Impulsivity is one major factor that has been suggested to contribute to the onset and maintenance of binge eating as well as obesity. Impulsivity is generally understood as a personality trait that comprises two components, according to Dawe and Loxton [9]: (a) reward sensitivity, i.e., the drive for appetitive or rewarding stimuli, and (b) rash-spontaneous behaviour, i.e., disinhibited behaviour without regard for the consequences. General trait impulsivity seems to be increased in obese people with or without BED and in people with other eating disorders showing binge eating behaviour [10], [11]. Moreover, first experimental studies of obese participants with BED indicate that both components of impulsivity are more increased towards disorder-specific food stimuli than in general, i.e., towards nonfood stimuli [12], [13]. Furthermore, there is first evidence that obese people with and without BED show different genetic expressions concerning reward sensitivity [14], and on a behavioural level, we assume in our recent review [15] that obese people with BED show even more pronounced deficits than obese people without BED in both food-related reward sensitivity and rash-spontaneous behaviour. This leads to the assumption that BED represents an enhanced neurobehavioural phenotype of obesity [16].

However, evidence in people with BED concerning food-related reward sensitivity and rash-spontaneous behaviour is still sparse, and studies usually do not discriminate between obese people with and without BED [15]. To the best of our knowledge, no study thus far has investigated both components of food-related impulsivity in obese people with BED. To fill this gap, we used eye tracking methodology, which offers a direct measure of cognitive and motivational processes with high temporal resolution. Eye tracking was previously used to investigate food-related reward sensitivity in overweight or obese people [17]–[19], but never in patients with diagnosed BED. These studies simultaneously presented food and nonfood stimuli and showed that overweight people perceive food as highly rewarding, as they initially fixated on food stimuli more often, gazed longer overall on food stimuli, or showed faster reaction times towards food stimuli than normal-weight control participants, although the results were not consistent. To measure rash-spontaneous behaviour, another well-established eye tracking paradigm is the antisaccade task, in which people are instructed to look away from a newly appearing neutral stimulus [20], [21]. This paradigm was widely used with simple geometric forms to explore impulse control in patients with different neurological disorders [20], [22], [23]. To perform such a voluntary antisaccade, participants have to inhibit the visual grasp reflex, the automatic reaction to direct the view towards the stimulus [21]. A directional error occurs when participants look towards the stimulus instead of looking away. Studies usually analysed directional errors of the first saccade to measure response inhibition, but some studies also analysed corrective behaviour in the second saccade which is shown to be impaired in some patient groups [20], [24]. Initial studies adapted this paradigm and used disorder-specific instead of neutral stimuli in patients with anxiety disorders [25] and with social drinkers [26], whereas we used food stimuli in the antisaccade paradigm for the first time.

In the present study, we explored both components of impulsivity in obese participants with BED (BED+) in two separate experiments using identical stimulus material. To control for the influence of both overweight and eating disorder, we investigated two control groups: obese participants without BED (BED−) with matched body mass index (BMI) and healthy normal- weight control participants (NWC). As women suffer more often from BED than men [7] and to avoid gender effects, we included women exclusively in our study. In experiment 1, we measured food-related reward sensitivity in a free exploration paradigm by presenting pairs of food vs. nonfood stimuli. We expected BED+ participants to initially fixate more often on food stimuli and to spend more time overall gazing on food stimuli in comparison with BED− and NWC participants. Additionally, this bias towards food stimuli should also be increased in BED− participants in comparison with NWC participants. In experiment 2, we conducted a modified antisaccade paradigm to measure rash-spontaneous behaviour towards food and nonfood stimuli. We expected the BED+ group to show more directional errors on the first and second saccade compared with the BED− and NWC groups. Again, the error rate should also be increased in BED− compared with NWC and more pronounced in food compared with nonfood stimuli. Additionally, we expected that BED+ participants would perceive food stimuli as more pleasant than both other groups and that eye tracking performance in both experiments would correlate with self-reported trait impulsivity.

## Materials and Methods

### Ethics Statement

The ethics committee of the medical faculty of Eberhard Karls University Tübingen and of the University Hospital Tübingen approved the study. All participants gave written informed consent.

### Participants

We assessed 27 overweight or obese women (BMI 27–45 kg/m^2^) with a diagnosis of BED or subthreshold BED (3 participants with subthreshold diagnosis) according to DSM-IV (BED+). As controls, 26 age- (+/−5 years) and weight-matched (+/−5 BMI points) overweight or obese women without BED (BED−) and 25 age-matched healthy, normal-weight (BMI 19–25 kg/m^2^) women (NWC) were assessed. Exclusion criteria in all groups comprise impaired and non-corrected vision, somatic diseases or medication influencing weight or eating behaviour, pregnancy or lactation, psychotropic medication use except antidepressants, and according to DSM-IV, psychosis, bipolar I disorder or substance addiction, and any eating disorder in both control groups. We excluded one BED+ participant due to uncorrectable vision and another BED+ participant due to not fulfilled (subthreshold) BED diagnosis, resulting in 25 BED+ participants. Sample characteristics are shown in table 1.

**Table 1:** Mean (± standard deviation) of sample characteristics in each study group.

	BED+(*n = *25)	BED−(*n = *26)	NWC (*n = *25)	*p*	post hoc group differences
Age (years)	39.7 (±11.7)	39.9 (±12.6)	39.4 (±11.8)	.989	–
BMI (kg/m^2^)	35.4 (±5.6)	35.4 (±5.4)	22.5 (±1.6)	.000	BED+, BED−>NWC
Binge eating frequency^a^	12.0 (±9.8)	0	0	.000	BED+>BED−, NWC
Eating pathology^b^	3.0 (±0.9)	1.3 (±1.0)	0.3 (±0.3)	.000	BED+>BED−>NWC
BIS-11 sum score	66.9 (±10.0)	60.5 (±9.1)	60.6 (±7.8)	.019	BED+>BED−, NWC
BIS-11 attention	11.1 (±3.1)	9.0 (±1.8)	9.2 (±2.2)	.023	BED+>BED−, NWC
BIS-11 self-control	14.7 (±3.1)	12.8 (±3.2)	12.1 (±2.4)	.009	BED+>NWC
BAS total score	3.1 (±0.4)	3.0 (±0.3)	3.0 (±0.3)	.388	–
BAS reward responsiveness	3.3 (±0.4)	3.1 (±0.4)	3.1 (±0.3)	.305	–
Depression^c^	15.6 (±11.9)	4.8 (±6.4)	1.7 (±2.1)	.000	BED+>BED−, NWC
Hunger ratingd, e	1.5 (±1.8)	1.5 (±1.8)	1.1 (±1.3)	.996	–

BAS, Behavioural Activation System scale; BDI II; Becks Depression Inventory II; BED+, overweight or obese women with binge eating disorder; BED−, overweight or obese women without binge eating disorder; BIS-11, Baratt Impulsiveness Scale; EDE-Q, Eating Disorder Examination Questionnaire; NWC, normal-weight control women.

a self-reported in the last four weeks according to EDE-Q item 15.

b self-reported according to EDE-Q total score.

c self-reported according to BDI II sum score.

d assessed after a standardised meal before testing on visual analogue scales ranging from 0 to 10 cm.

e *n* = 24 in the BED+ group due to missing data in one participant.

### Stimuli

The stimulus material consisted of 24 coloured low- to high-caloric food stimuli and 24 nonfood stimuli depicting everyday objects. The food and nonfood stimuli were closely matched for physical properties such as colour, brightness, and contrast; pretested for complexity, valence, and arousal and were previously used in fMRI studies of obese participants [27].

### Experimental Paradigms

#### Experiment 1: free exploration paradigm

**Figure 1:**
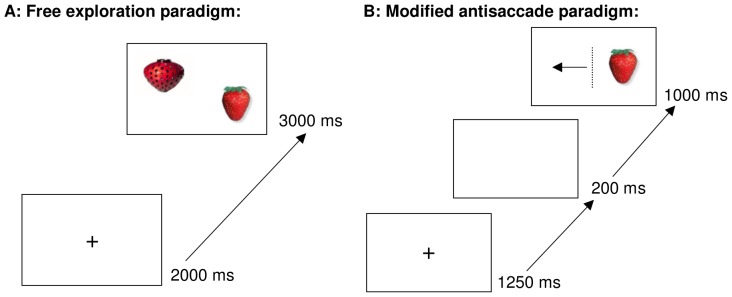
Schematic presentation of the free exploration paradigm (A) and the modified antisaccade paradigm (B). Panel A: Each of the two stimuli (food vs. nonfood) had a height of 8.2 cm and a width of 12 cm and were located with the midmost corner 0.8 cm away from the centre of the screen. The position of the stimuli (upper vs. lower and left vs. right quadrant) was balanced. Panel B: The stimuli (food or nonfood) had a height of 8.7 cm and a width of 11.8 cm and were located 3.5 cm from the centre of the screen. The position of the stimuli (left vs. right) was randomised.

To assess reward sensitivity, 24 pairs of food and nonfood stimuli were randomly presented, and participants were instructed to freely explore them as if they were watching TV (see fig. 1A). Before the presentation of a stimulus pair, a fixation cross was displayed in the middle of the screen and participants were asked to fixate on this cross between trials. This paradigm has already been used with food stimuli in people with anorexia nervosa [28] and overweight people [29]. The initial fixation position on a stimulus represents automatic attention orienting, and the total gaze duration represents conscious and ongoing attention, with both measures indicating a preference towards a stimulus and the activation of the brain reward system [17], [28], [29].

#### Experiment 2: modified antisaccade paradigm

To assess rash-spontaneous behaviour, a food or nonfood stimulus appeared randomly on the left or right side of the screen, and participants were instructed to look away from this stimulus in the opposite direction as fast as they could (see fig. 1B). Stimuli were presented twice, resulting in 96 trials. Before the appearance of a stimulus, a fixation cross, followed by a blank screen, was displayed. The original antisaccade paradigm measures rash-spontaneous behaviour as brain areas that are associated with inhibitory processes are activated during performance [21], [30]. Accordingly, the modified antisaccade paradigm measures rash-spontaneous behaviour and, additionally, differentiates between disorder-specific rash-spontaneous behaviour towards food stimuli and in general, i.e., towards nonfood stimuli. The percentage of directional errors in the first saccade correlates with trait impulsivity and contributes to the same construct as other response inhibition paradigms [20], [31]. The analysis of directional errors in the second saccade, especially corrective gaze behaviour after directional errors in the first saccade, additionally addresses deficits in response generation and self-monitoring [20], [32].

### Procedure

At the first study appointment, we assessed height, weight, socioeconomic variables, and diagnosed BED as well as comorbid disorders using the Structured Clinical Interview for Axis I Disorders (SCID-I) [33]. Additionally, participants reported eating pathology using the Eating Disorder Examination Questionnaire (EDE-Q) [34] and depression using Becks Depression Inventory (BDI II) [35]. Participants were instructed to fast overnight before they came to the second study appointment at 8.30 a.m., which started with a standardised breakfast to control for hunger. After a 13-point calibration to adapt the eye tracker to the individual properties of the participants, experiments 1 and 2 were conducted. Before and after the experiments, participants rated their hunger on visual analogue scales ranging from 0 cm (not hungry) to 10 cm (extremely hungry). Afterwards, a computer-based rating of the pleasantness of all stimuli was conducted, with a scale ranging from −5 (extremely unpleasant) to +5 (extremely pleasant). Additionally, participants completed two questionnaires measuring trait impulsivity: the Baratt Impulsiveness Scale (BIS-11) [36] and the BIS/BAS Scale, according to Gray’s behavioural inhibition and activation systems [37]. According to several factor analyses [9] and one correlational study with the classical antisaccade paradigm [31], the BAS scale from the BIS/BAS measures predominantly reward sensitivity, and the BIS-11 assesses primarily rash-spontaneous behaviour. Therefore, we investigated the BAS total score and especially the BAS subscale reward responsiveness, as this scale seems nearest to reward sensitivity. From BIS-11, we used the sum score as well as the subscales attention and self-control to specifically investigate rash-spontaneous behaviour. Internal consistency of the scales in the present sample was overall in an acceptable to good range, with *α* = .82 (*n* = 70) for BIS-11 sum score, *α* = .63 (*n* = 75) for BIS-11 attention, *α* = .70 (*n* = 76) for BIS-11 self-control, *α* = .67 (*n* = 75) for BAS total score and *α* = .42 (*n* = 76) for BAS reward responsiveness.

### Apparatus

Eye movements were recorded with the IViewX Hi-Speed eye tracking system [38] with a 500 Hz sampling rate, 0.25°–0.5° gaze position accuracy, and IViewX 2.8 software. The eye tracking tower with the chin and forehead pad was arranged 60 cm in front of a 19-inch computer screen with a resolution of 1280×1024 pixels.

### Eye Movement Data

Raw data for each participant was analysed with BeGaze 3.0 [39] using the velocity-based default algorithms that define fixations and saccades. Afterwards, data was reduced and aggregated across participants. Trials in both experiments were excluded if participants did not fixate on the cross at the onset of the trial and if data was not recorded due to technical problems.

In experiment 1, the data was exclusively analysed for two predefined areas of interest (AOIs) that display the food and nonfood stimuli. Participants with two standard deviations (*SD*) above mean excluded trials or two *SDs* below the mean total gaze duration on both AOIs were excluded from data analysis due to lowered data quality, so that sample size was reduced to *n* = 70 (BED+*n* = 24, BED−*n* = 24, NWC *n* = 22). Two variables for hypothesis testing were defined: the percentage of a) the initial fixation position on each AOI and b) the total gaze duration on each AOI.

In experiment 2, saccades starting below 80 ms or above 700 ms after target onset were excluded, as they are considered as anticipatory or delayed [25], [40]. Participants with more than 50% of excluded trials or two *SDs* above the mean first saccade error rate per group were excluded from data analysis, so that sample size was reduced to *n* = 63 (BED+*n* = 23, BED−*n* = 19, NWC *n* = 21). Two variables for hypothesis testing were defined: the percentage of directional errors in food vs. nonfood trials on a) the first saccade (first saccade errors) and b) the second saccade (second saccade errors). We additionally analysed the percentage of sequential errors with directional errors on the first and the second saccade, i.e., when participants failed to correct errors.

### Statistical Analysis

Data was analysed with IBM SPSS Statistics 20.0. Sample characteristics were analysed with analyses of variance (ANOVA) and with the Kruskal-Wallis test, Mann-Whitney U test or Pearson’s Chi-Square test, if the requirements for ANOVA were violated. Eye tracking data was analysed with repeated measure ANOVAs with stimulus (food, nonfood) as within subject factor and group (BED+, BED−, NWC) as between subject factor. In a second step, we computed bias scores (Δ food – nonfood stimuli) for each eye tracking variable to compute planned contrasts in univariate ANOVAS with group as between subject factor. Additionally, Pearson correlations (*r*) as parametric and Spearman correlations (*φ*) as nonparametric tests were computed to analyse relationships between these bias scores and self-reported impulsivity. Statistical significance was determined at *α* = .05.

## Results

### Sample Characteristics

Sample characteristics are presented in table 1. Groups were comparable with respect to educational status, marital status, and nationality (all *p*>.05). 8 participants (11% of the whole sample) currently used antidepressants, with 6 of them using selective serotonergic reuptake inhibitors (SSRIs). BED+ participants used antidepressants more often than BED− and NWC together (6 BED+ participants, i.e., 24% and 2 BED− participants, i.e., 4.2%; *Χ* ^2^(1) = 6.63, *n* = 73, *p* = .010).

### Experiment 1: Free Exploration Paradigm

**Figure 2:**
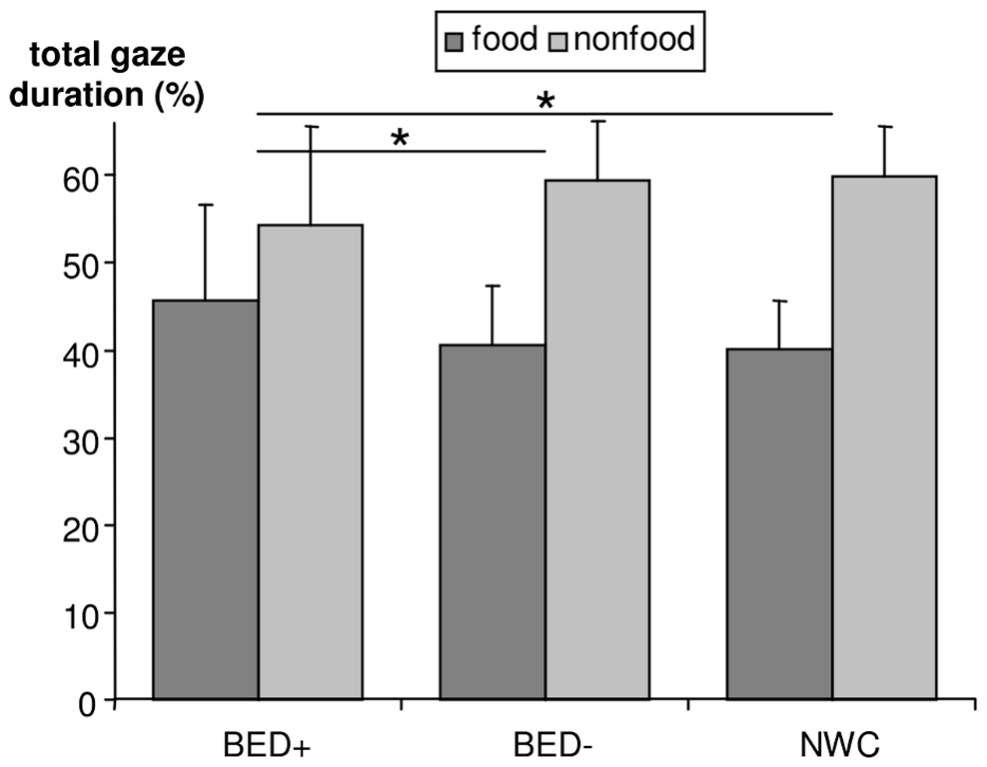
The percentage of total gaze duration on food vs. nonfood stimuli. Figures showing means and standard deviations. A significant stimulus main effect (*p*<.05) and a group × stimulus interaction with significant differences between BED+ and BED− and BED+ and NWC (both *p*<.05, marked as *) in planned contrasts were found. BED+, overweight or obese women with binge eating disorder; BED−, overweight or obese women without binge eating disorder; NWC, normal-weight control women.

Regarding the percentage of the initial fixation position on each AOI, a significant main effect on stimulus elapsed (*F*[1,67] = 19.9, *p*<.001, *e* ^2^ = .23; *M* = 54.8, *SD* = 8.9 in food stimuli, *M* = 45.2, *SD* = 8.9 in nonfood stimuli), indicating that all groups prefer to initially fixate on food stimuli. Analysis of the percentage of the total gaze duration shows a significant main effect for stimulus (*F*[1,67] = 64.5, *p*<.001, *e* ^2^ = .49; *M* = 42.2, *SD* = 8.5 in food stimuli, *M* = 57.8, *SD* = 8.5 in nonfood stimuli) and a significant group × stimulus interaction (*F*[2,67] = 3.3, *p* = .04, *e* ^2^ = .09). Planned contrasts with the bias score of the percentage of total gaze duration show that BED+ participants significantly differ from BED−(*t*[67] = −2.1, *p = *.036) and from NWC (*t*[67] = 2.3, *p = *.025) participants, whereas BED− and NWC participants do not differ from each other. As illustrated in figure 2, these results indicate that nonfood stimuli are fixated longer overall than food stimuli, but BED+ participants allocate more attention towards food stimuli than both other groups.

### Experiment 2: Modified Antisaccade Paradigm

**Figure 3:**
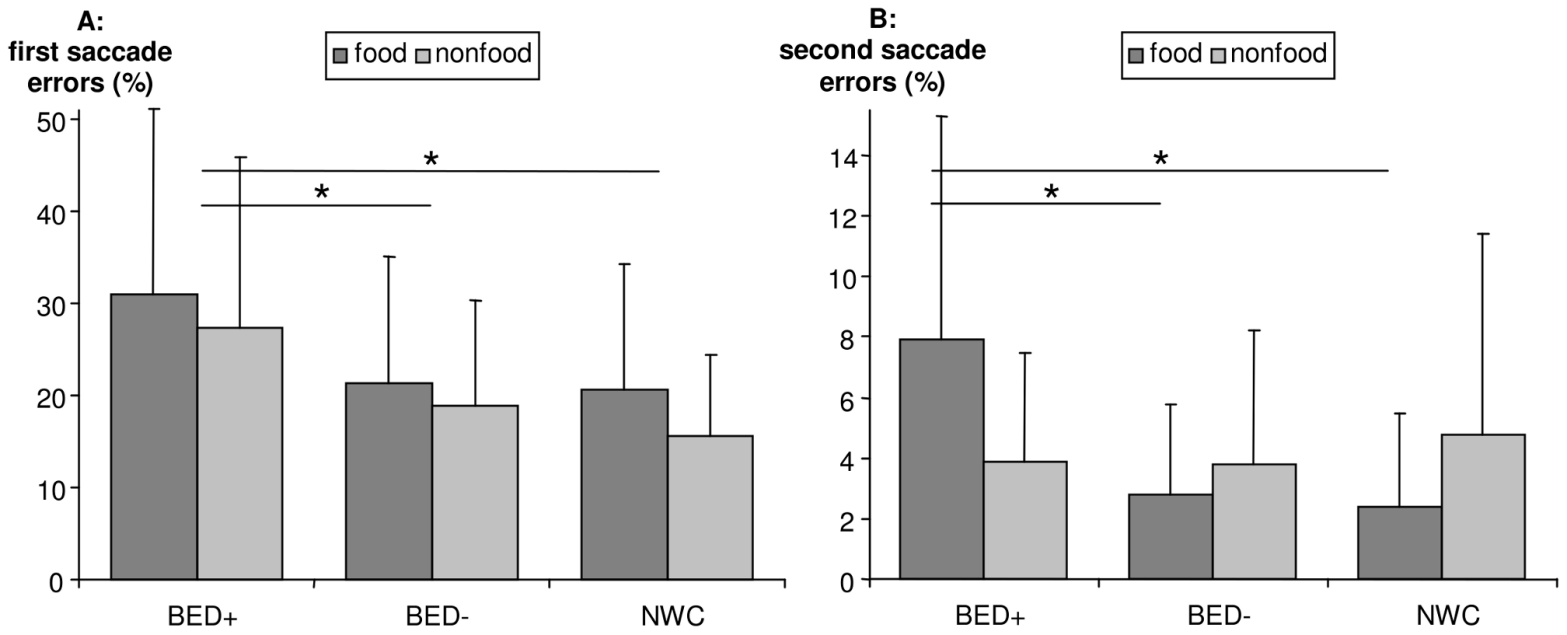
The percentage of first saccade errors (A) and the percentage of second saccade errors (B). Figures showing means and standard deviations. Panel A: Significant group and stimulus main effects (both *p*<.05) were found with significant differences between BED+ and BED− and BED+ and NWC in planned contrasts for the group effect (both *p*<.05, marked as *). Panel B: A significant group × stimulus interaction (*p*<.05) was found with significant differences in planned contrasts between BED+ and BED− and BED+ and NWC (both *p*<.05, marked as *). BED+, overweight or obese women with binge eating disorder; BED− overweight or obese women without binge eating disorder; NWC, normal-weight control women.

The ANOVA for the percentage of first saccade errors yielded a significant main effect for stimulus (*F*[1,60] = 9.6, *p = *.003, *e* ^2^ = .14; *M* = 24.7, *SD* = 16.8 in food stimuli, *M* = 20.8, *SD* = 14.6 in nonfood stimuli) and for group (*F*[2,60] = 3.8, *p* = .028, *e* ^2^ = .11; *M* = 29.3, *SD* = 18.7 in BED+, *M* = 20.1, *SD* = 12.0 in BED−, *M* = 18.0, *SD* = 10.4 in NWC). Planned contrasts for the group factor show that BED+ significantly differ from BED− (*t*[60] = −2.06, *p = *.04) and from NWC (*t*[60] = 2.6, *p = *.013), whereas BED− and NWC do not differ from each other. Thus, all groups make more first saccade errors in food trials than in nonfood trials and BED+ make more first saccade errors in both stimulus conditions in comparison with both other groups (see fig. 3A). For the percentage of second saccade errors, the ANOVA yielded a significant group × stimulus interaction (*F*[2,60] = 7.3, *p* = .001, *e* ^2^ = .20). Planned contrasts with the bias score of second saccade errors show that BED+ make more second saccade errors than BED− (*t*[60] = −2.8, *p = *.008) and NWC (*t*[60] = 3.6, *p = *.001) in food but not in nonfood trials (see fig. 3B).

Analysis of the percentage of sequential errors (s. table 2) shows first, a significant group effect (*F*[2,60] = 4.3, *p* = .018, *e* ^2^ = .13) with planned contrasts yielding a significant difference between BED+ and BED− (*t*[60] = −2.3, *p* = .028) as well as NWC (*t*[60] = 2.7, *p* = .009). Second, a significant group × stimulus interaction emerged (*F*[2,60] = 4.9, *p* = .011, *e* ^2^ = .14) with planned contrasts for the bias score yielding a significant difference between BED+ and BED− (*t*[60] = −2.6, *p* = .011) as well as NWC (*t*[60] = 2.7, *p* = .009). Thus, BED+ participants made more sequential errors than BED− and NWC participants in both stimulus conditions, especially in food trials.

**Table 2:** Mean (± standard deviation) for the percentage of sequential errors.

	BED+(*n = *23)	BED− (*n = *19)	NWC (*n = *21)
Overall sequential errors (%)	4.1 (±4.1)	1.9 (±2.2)	1.5 (±1.8)
Sequential errors in food trials (%)	5.6 (±6.6)	1.6 (±2.1)	1.3 (±2.2)
Sequential errors in nonfood trials (%)	2.4 (±2.7)	2.2 (±3.3)	1.9 (±3.6)

Significant differences in planned contrasts for the group main effect (see overall sequential errors) as well as for the group × stimulus interaction were found between BED+ and BED− and BED+ and NWC (each *p*<.05). BED+, overweight or obese women with binge eating disorder; BED−, overweight or obese women without binge eating disorder; NWC, normal-weight control women.

### Food and Nonfood Ratings

In all groups, food stimuli were rated as more pleasant than nonfood stimuli (*F*[1,73] = 40.2, *p = *.000; *e* ^2^ = .36; *M* = 2.1, *SD* = 2.6 in food stimuli; *M* = −0.4, *SD* = 1.8 in nonfood stimuli). Groups did not differ in ratings of nonfood stimuli (*p*>.05; *M* = −0.7, *SD* = 1.9 in BED+, *M* = −0.2, *SD* = 1.8 in BED−, *M* = −0.3, *SD* = 1.8 in NWC), but BED+ participants rated food stimuli as more pleasant than both other groups did (*F*[2,73] = 3.47, *p = *.036; *M* = 3.2, *SD* = 2.7 in BED+, *M* = 1.5, *SD* = 2.8 in BED−, *M* = 1.6, *SD* = 2.0 in NWC) with planned contrasts showing significant differences between BED+ and BED− (*t*[73] = −2.3, *p* = .022) as well as NWC (*t*[73] = 2.2, *p* = .030).

### Correlational Analyses

Two-tailed correlations show that the bias score of the initial fixation position does not correlate with BIS-11 or BAS subscales, whereas the bias score of the total gaze duration correlates with the BAS reward responsiveness subscale (*φ* = .24, *p* = .043, *n* = 70). The bias score of first saccade errors correlates negatively with the BAS reward responsiveness subscale (*φ* = −.35, *p* = .005, *n* = 63), whereas the bias scores of second saccade errors and sequential errors each correlate with the BIS-11 subscale of self-control (*φ = *.27, *p* = .031, *n* = 63 and *φ = *.27, *p* = .035, *n* = 63 respectively). BIS-11 sum score and BAS total score did not correlate with these five bias scores (*p*>.05). Additionally, exploratory correlations of the bias scores with BDI II sum score show no significant correlations after Bonferroni correction for multiple testing (all *p*>.05).

## Discussion

This is, to our knowledge, the first eye tracking study to explore information processing of food stimuli in binge eating disorder (BED). We particularly investigated both components of food-related impulsivity in BED in one study – reward sensitivity and rash-spontaneous behaviour [9]. To explore these two components, we compared data from two paradigms in overweight or obese patients with BED (BED+) with two accurately matched control groups, one age- and BMI-matched group without BED (BED−) and one age-matched and normal-weight control group (NWC). Both the established free exploration paradigm and the newly modified antisaccade paradigm have been proven useful to investigate impulsivity: They could differentiate between BED+ and controls, and they correlated with trait impulsivity even in a satiated state and by using rather slight stimuli (pictures of low- and high-caloric foods). Compared with both control groups, BED+ allocated longer visual attention to food stimuli, which indicates increased food-related reward sensitivity. Moreover, those in the BED+ group had more difficulties voluntarily suppressing first saccades to peripheral cues, indicating generally increased rash-spontaneous behaviour, and especially towards food stimuli in second saccades and concerning sequential errors. These results support our main hypothesis that BED+ individuals show increased reward sensitivity and rash-spontaneous behaviour towards food stimuli. However, the second part of our hypothesis, i.e., BED− also show increased impulsivity concerning both impulsivity components, was not supported.

More detailed regarding reward sensitivity, BED+ participants gazed longer at food stimuli than BED− and NWC participants. This is in line with our hypothesis concerning increased ongoing and conscious attention allocation towards food stimuli in BED+. This bias can be interpreted as increased food-related reward sensitivity, as increased pleasantness ratings of food stimuli in the BED+ group compared with both control groups additionally support this interpretation. One surprising result concerning total gaze duration is that all groups gazed longer on nonfood than on food stimuli. This might be due to the high semantic complexity of nonfood stimuli in this study, which might have led to an increased processing time in order to determine the content of the nonfood stimuli. Concerning initial fixation position, we did not find the expected group differences because all groups located their attention primarily on food stimuli. Thus, the hypothesis concerning increased early attention orienting towards food stimuli in BED+ individuals is not supported. This might be due to a biological advantage of recognising food, which causes all groups to fixate on food stimuli first [41] and fits to our results that all groups rated food stimuli as more pleasant than nonfood stimuli. Taken together, our results concerning reward sensitivity are in line with first experimental studies investigating BED, e.g., using fMRI and EEG, which show increased reward sensitivity in BED+ compared with BED− or NWC participants [12], [42], [43].

Concerning rash-spontaneous behaviour, all groups made more first saccade errors in food trials than in nonfood trials and the BED+ group showed more first saccade errors in both stimulus conditions in comparison with both other groups. The BED+ group made more second saccade errors compared with both other groups in food but not in nonfood trials and more sequential errors in both stimulus conditions, especially in food trials. Thus, on first saccades, BED+ participants initially show generalised deficits in suppressing an undesirable reaction, and concerning second saccades and sequential errors, they show increased disinhibited behaviour, especially concerning food stimuli. Hence, even if BED+ individuals succeed in inhibiting the first saccade towards a novel-appearing food stimulus, they often fail to maintain this inhibition on the second saccade, where turning towards the desired stimulus could no longer be suppressed. Concerning sequential errors, if BED+ participants have already had a faulty executed saccade, they have remarkable difficulties disentangling their attention and thus, are not that able to correct errors easily. This extended response inhibition deficit could be due to the attention-grabbing properties of food stimuli for BED+ participants. Taken together, BED+ participants might initially show generally increased tendencies for rash-spontaneous behaviour that is, once more, pronounced in confrontation with food cues in later stages of processing. Earlier experimental studies have also suggested inhibitory deficits towards food stimuli in BED+ participants in comparison with NWC or BED− participants, although these studies did not explore general rash-spontaneous behaviour [44] or did not use a normal-weight control group [13].

We additionally explored associations between trait impulsivity and eye tracking performance. Participants who demonstrated higher reward responsiveness in self-reports tended to gaze longer on food stimuli according to the bias score of the total gaze duration. This underpins the validity of the free exploration paradigm in assessing impulsivity, especially reward sensitivity. The error bias score in first saccades decreased with higher reward responsiveness. Thus, increased reward sensitivity seems to protect people from erroneous behaviour in first saccades towards food stimuli, which might be explained by the high motivation of generally high reward-seeking individuals to perform tasks as well as possible. Moreover, the error bias scores in second saccade errors and sequential errors increased with lower self-control and therefore measure impulsive, disinhibited behaviour with loss of control concerning one’s own actions. Hence, specific facets of trait impulsivity measured with subscales of both impulsivity questionnaires seem to be related to the collected gaze parameters, although the BAS subscale reward responsiveness featured low internal consistency and the BIS-11 sum score and BAS total score in general did not correlate. As performance in experimental impulsivity paradigms generally shows no or only weak associations with trait impulsivity [11], [31], the demonstrated correlations are remarkable.

Nevertheless, some limitations of the presented study should be considered. First, cognitive and motivational processing can occur independently from actual eye movements. Second, BED− participants did not differ unexpectedly from NWC participants, whereas two eye tracking studies [17], [19] indicate increased impulsivity in obese people. However, these studies did not control for BED diagnosis, and the reported effects could be due to included participants with BED [15]. Otherwise, we used high and low-caloric stimuli which may not be sufficient to detect increased impulsivity in BED− subjects, as the reward system shows stronger activity towards high-caloric food in obese subjects [17], [27], [45]. Therefore, the efficacy of our eye tracking paradigms may have been increased if only high-caloric food stimuli and nonfood stimuli with less semantic complexity had been used. Third, we also included participants with depressive symptoms and using antidepressant medication, especially in the case of BED+ participants. Although depression did not correlate with the eye tracking measures, and antidepressants such as SSRIs do not affect oculomotor processes, according to a review by Reilly and colleagues [46], we cannot rule out that this may have had an impact upon our results. Last, we did not assess a fourth group comprised of normal-weight people with BED. The inclusion of such a group might help to further unravel the association of food-related impulsivity with binge eating behaviour or obesity.

Altogether, our results clearly support the idea of increased food-related reward sensitivity and rash-spontaneous behaviour in patients with BED. Hence, our results support the view that BED represents an enhanced neurobehavioural phenotype of obesity [16]. Additionally, the reported eating behaviours of BED patients fit well with increased reward sensitivity towards food stimuli, which might appear as a strong desire or craving for food, and increased rash-spontaneous behaviour strongly resembles the experienced loss of control while bingeing. Thus, specific interventions taking both impulsivity components into account might enhance the effectiveness of psychotherapy in obese patients with BED. For example, cognitive behavioural interventions such as cue exposure and response control [47] are closely linked to food-related impulsivity and might be expanded as a training programme that is focused particularly on food-related impulsivity. Moreover, training programmes with both of the presented eye tracking paradigms might lead to decreased impulsivity and, in turn, reduce binge eating episodes [48].
